# Some Aspects and Convergence of Human and Veterinary Drug Repositioning †

**DOI:** 10.3390/molecules29184475

**Published:** 2024-09-20

**Authors:** Patrik Mag, Melinda Nemes-Terényi, Ákos Jerzsele, Péter Mátyus

**Affiliations:** 1 Department of Pharmacology and Toxicology, University of Veterinary Medicine, István Street 2, 1078 Budapest, Hungary; mag.patrik@univet.hu (P.M.); terenyi.melinda@univet.hu (M.N.-T.); jerzsele.akos@univet.hu (Á.J.); 2 National Laboratory of Infectious Animal Diseases, Antimicrobial Resistance, Veterinary Public Health and Food Chain Safety, University of Veterinary Medicine, István Street 2, 1078 Budapest, Hungary

**Keywords:** human and veterinary drug innovation, drug repositioning, repositioning of COX inhibitors, one health drug repositioning

## Abstract

Drug innovation traditionally follows a de novo approach with new molecules through a complex preclinical and clinical pathway. In addition to this strategy, drug repositioning has also become an important complementary approach, which can be shorter, cheaper, and less risky. This review provides an overview of drug innovation in both human and veterinary medicine, with a focus on drug repositioning. The evolution of drug repositioning and the effectiveness of this approach are presented, including the growing role of data science and computational modeling methods in identifying drugs with potential for repositioning. Certain business aspects of drug innovation, especially the relevant factors of market exclusivity, are also discussed. Despite the promising potential of drug repositioning for innovation, it remains underutilized, especially in veterinary applications. To change this landscape for mutual benefits of human and veterinary drug innovation, further exploitation of the potency of drug repositioning is necessary through closer cooperation between all stakeholders, academia, industry, pharmaceutical authorities, and innovation policy makers, and the integration of human and veterinary repositioning into a unified innovation space. For this purpose, the establishment of the conceptually new “One Health Drug Repositioning Platform” is proposed. Oncology is one of the disease areas where this platform can significantly support the development of new drugs for human and dog (or other companion animals) anticancer therapies. As an example of the utilization of human and veterinary drugs for veterinary repositioning, the use of COX inhibitors to treat dog cancers is reviewed.

## 1. Introduction

Human and veterinary drug innovation takes place from discovery through a multi-stage pathway to marketing a drug. Two main phases can be distinguished: the first is the research and development preclinical phase, which is followed by the clinical development phase. With the successful completion of the clinical phase, it is possible to submit a new drug application (NDA), which is reviewed by the regulatory authority. If all conditions (efficacy, safety, etc.) are met, the drug is allowed to be placed on the market (this is also called regulatory approval or authorization). The long-term safety of the drug’s use is also monitored (post-marketing monitoring).

Traditionally, the de novo (from the beginning to the end) drug innovation strategy is followed. It began with the design of new molecules, mainly with intuitive and experience-based chemistry until the 1990s. In more recent decades, the expanding support of various computational (in silico) molecular modelling methods and the utilization of increasingly detailed knowledge of the biological target have come into play. In addition, especially since the turn of the millennium, another approach, drug repositioning, has also gained strategic importance.

This paper aims to provide an overview of human and veterinary drug innovation, with a particular focus on drug repositioning. It discusses the pros and cons of drug repositioning in human and veterinary medicine, as well as their similarities and differences. It also presents the important role of the repositioning strategy in one of the most challenging therapeutic areas: oncology drug therapy. More specifically, the treatment of oncological diseases in dogs with the repositioning of COX inhibitors is examined.

These topics are discussed in the following six main sections:

2. De novo drug innovation:
  2.1. Current landscape;
  2.2. Human and veterinary de novo drug innovation.
3. Drug repositioning:
  3.1. General overview;
  3.2. Human and veterinary drug repositioning.
4. Business aspects of drug innovation:
  4.1. Patent-based market exclusivity:
    4.1.1. New Chemical Entity;
    4.1.2. Second medical use.
  4.2. Regulatory-based market exclusivity;
  4.3. The role of off-label use in drug repositioning.
5. Drug repositioning for oncological diseases:
  5.1. Current aspects of drug therapy in oncology;
  5.2. Drug repositioning of COX-2 inhibitors for the treatment of dog cancers.
6. One Health approach to drug repositioning.
7. Conclusion and recommendations.

The second section describes de novo drug innovation. In the third section, the potential advantages and limitations of drug repositioning, the pharmacological basis, and the pool of repositioning candidates are discussed. The fourth section reviews the business aspects of drug innovation, particularly drug repositioning. In the fifth section, the role of drug repositioning and its current place in the drug therapy of oncological diseases is summarized. To illustrate the important role of drug repositioning in veterinary oncology, the repositioning of anti-inflammatory COX-2 inhibitors for the therapy of canine cancers is systematically reviewed. In the sixth section, the One Health approach to drug repositioning is described. In the seventh section, policies and measures to improve the productivity of drug repositioning are proposed. Finally, we conclude with our perspective on drug repositioning.

## 2. De Novo Drug Innovation

### 2.1. Current Landscape

The de novo drug innovation path leads to a new drug whose active ingredient has not been previously authorized for marketing. For human drugs, the entire process to commercialization takes 10–15 years, and this has barely changed in the last two decades. However, during this same period, the cost has continually risen. According to the analysis by DiMasi et al., in 2013, the out-of-pocket cost per approved new compound was estimated at USD 1395 billion and the capitalized cost at USD 2558 billion [1]. These figures are consistent with those published by Deloitte [2]. Another cost analysis by Prasad et al. calculated a significantly lower cost of USD 648.0 million based on newly approved oncology drugs over a decade ending in 2015 [3]. The difference between the figures of DiMasi and Prasad can be explained by differences in data sources and methodologies, but the cost of developing a new drug is still significant.

It is perhaps surprising that the efficiency of innovation has not improved continuously and substantially, despite the increasing financial expenditures. Instead, it has fluctuated from year to year. Innovation productivity consists of several components. Examining the quality of the output, some particularly favorable developments can be recognized. First among these are the new disease-modifying drugs introduced in certain therapeutic areas. The pharmaceutical portfolio, in general, has also seen some quality renewal, with advances in many areas of drug therapy that have improved patients’ quality of life and increased their lifespan. High-impact examples include COVID-19 vaccines, pharmacological therapy for HIV, and many very effective biologics. On the other hand, there are still diseases for which no satisfactory therapy is available, including those affecting a large number of patients now and in the future (e.g., CNS, immune-mediated, and oncological diseases). These diseases represent a pressing burden for healthcare.

The poor success rate of drug candidates is especially alarming. The efficiency of the clinical development phase from phase I clinical trials to registration is very low, usually less than 10% [4,5]. In certain therapeutic areas, the success rate is even significantly lower (e.g., 6% or less in oncology developments). The number of new medicines fluctuates, for example, the FDA (Food and Drug Administration, USA)’s Center for Drug Evaluation and Research (CDER) approved 21, 45, 37, and 55 novel drugs in 2010, 2015, 2022, and 2023, respectively [6]. A comprehensive analysis by Deloitte, based on 12 companies between 2010 and 2019, also confirms a fluctuation in pharmaceutical innovation [7].

Thus, the overall impression of the innovation landscape is not exactly encouraging. Pharmaceutical innovation has actually fallen short of expectations [8]. The emerging spectacular and revolutionary scientific and technological achievements of the 2000s have only partially translated into innovation in human pharmaceuticals.

### 2.2. Human and Veterinary De Novo Drug Innovation

The goal of veterinary and human drug innovation is ultimately the same: the commercialization of a new drug. The main stages (or phases) of both de novo innovation paths share common characteristics but also exhibit differences [9,10]. In the discovery phase of veterinary drug innovation, the same molecular design principles and methods are used to identify the lead compound and optimize it into a drug candidate. However, the formal preclinical and clinical development phases are quite different, warranting specific terminology for these phases in veterinary drug innovation [11]. The final stop on the innovation journey for new veterinary medicinal products is approval by the FDA’s Center for Veterinary Medicine (CVM) and its European counterpart, the European Medicines Agency (EMA), according to current laws and regulations [12,13,14].

In the human preclinical development phase, the drug candidate is tested in various preclinical models to provide pharmacodynamic, pharmacokinetic, and safety evidence for the approval of phase I clinical trials. In favorable cases, phase I trials are followed by phase II and III studies. However, in the veterinary preclinical development phase, the drug candidate is also tested in pharmacokinetic, toxicological, target animal safety, user safety, and environmental safety studies; then, in the clinical phase, these are followed by the clinical trial(s) to finally gain regulatory approval. In some cases, a pilot study is followed by a pivotal study. Although the veterinary development phase seems simpler compared to the human preclinical and clinical phases, it also poses specific challenges, such as the complexity of attracting and involving patients and organizing clinical trials.

Several aspects must be highlighted to conclude the comparison of human and veterinary de novo drug innovations. The veterinary drug innovation pathway can also take a long time. The (pivotal) clinical qualification of the veterinary drug candidate is more reliable. Disqualifying properties can be recognized in the early stages of development; thus, the innovation success rate is generally much higher, whilst the cost is much lower (depending on the legal basis, the animal species, and the disease) than that of human innovation.

It could be expected that this favorable scenario would ultimately encourage greater investment, resulting in more new veterinary medicines. Surprisingly, both the investment and the number of new drugs are quite low [15]. Companies are apparently less motivated to invest in the innovation of veterinary drugs. This is likely because of the lower returns on investment due to the peculiar characteristics of the veterinary pharmaceutical market [16].

Undoubtedly, this attitude will change due to increased demand for animal medicines. Steadily increasing sales of companion and farm animal medicines and vaccines are expected over the next ten years [17]. Overall, the income–cost ratio of companies will increase, which should stimulate further interest in the area. Moreover, as in human medicine, there are unmet medical needs in the veterinary field (for definitions of unmet medical need by authorities, see Guidance for Industry Expedited Programs for Serious Conditions—Drugs and Biologics [18]—and PRIME: priority medicines [19]; for a holistic interpretation, see EFPIA [20]), and so, just as in human medicine, it is necessary to expand the veterinary medicine portfolio with new, animal-specific medicines.

The generally slow and modest developments in both human and veterinary drug innovation, along with growing business interest, are perhaps encouraging for the future. However, achieving much more ambitious goals (e.g., significantly higher success rates, new and better, even breakthrough, drugs for many therapeutic areas, including those with unmet medical needs) with the current human and veterinary pharmaceutical innovation strategy and methodological arsenal alone is quite uncertain. Thus, it is almost imperative to renew the de novo strategy and, in addition, to develop new drug innovation strategies. Drug repositioning can be one such strategy.

## 3. Drug Repositioning

### 3.1. General Overview

Drug repositioning can be simply and broadly defined as using a previously approved drug or a failed investigational drug candidate for a new indication different from the originally intended therapeutic indication (for terminology and definitions, see Langedijk et al. [21]). In 2018, new terms were introduced according to the relationship of the first and second medical use. Soft repositioning is when a drug is used in the same area (e.g., oncology), whereas in the treatment of very different diseases (e.g., psychiatry and oncology), it is known as hard repositioning [22].

In this paper, we uniformly use the term “drug repositioning” (or simply, repositioning) and narrow its scope. Namely, (i) we only examine cases of repositioning approved drugs, and (ii) we focus on new medical uses that are not obvious (non-obvious) from the original application. We understand that it is difficult to precisely define and sharply separate the non-obvious from the obvious (as in the case of patenting). It is therefore advisable that this qualification is conducted on a case-by-case basis. To illustrate our interpretation, the use of a certain oncology drug in another oncology therapy could be a therapeutic extension or even a non-obvious repositioning. Thus, the veterinary use of a human drug for the same therapeutic indication is clearly an extension, not a repositioning.

Drug repositioning has a long history, dating back to the second half of the last century. Its best known and textbook examples are predominantly based on serendipity. One of the earliest examples of repositioning is thalidomide, which was withdrawn and banned worldwide in the early 1960s because of its teratogenic effects which caused tragic birth defects in more than 10,000 babies. By a serendipitous observation, thalidomide was approved for the treatment of leprosy in 1998, then, through a systematic path, for multiple myeloma in 2006. Notably, it is also used for the treatment of various cancers in dogs.

Fundamentally, an unexpected pharmacological or clinical effect or behavior of a known drug was observed, which was then utilized for an independent new (second) therapeutic purpose. The pathway of the repositioned drug to the second indication was different from the de novo pathway, as it was much shorter, cheaper, and—for these reasons—less risky. Not surprisingly, there has been a systematic and organized effort to develop and exploit drug repositioning as an alternative drug innovation strategy.

We believe that two particularly valuable innovative concepts indirectly but significantly contributed to the emergence of the drug repositioning framework. The earlier was developed by Sir J.W. Black, Nobel Prize Winner in 1988 [23]: “The most fruitful basis for the discovery of a new drug is to start with an old drug”. Secondly, C.G. Wermouth developed the Selective Optimization of Side Activities (SOSAs) concept [24], which optimized a potentially useful side effect to utilize the drug for a new indication.

After the first comprehensive review of drug repositioning in 2004 [25], numerous original reports and reviews have been published presenting additional examples and outlining the advantages and disadvantages of drug repositioning. Although there was generally a positive balance encouraging the use of this approach (for a recent review, see Khambhati et al. [26]), there are also cautious and critical, almost skeptical, opinions on its value [27,28,29,30,31,32,33,34,35,36,37,38,39,40].

The most important advantage of drug repositioning is that it can be significantly shorter (3–7 years), cheaper (approximately USD 300 million, saving up to 85% of de novo innovation costs), and more likely to succeed than de novo innovation [36]. Many safety, pharmacokinetic (PK), and pharmacodynamic (PD) features of the repositioning candidate (approved previously for the first medical use) were explored in clinics with long-term observations and found suitable for the first (original) therapeutic use. Therefore, the probability of failure for the second medical application, especially for safety reasons, is lower than for a de novo drug candidate, whose frequent failure is often associated with unacceptable safety issues [41,42].

Nonetheless, it should be noted that the PK/PD features of the original application are less predictable in the new development if the formulation, dose, route of administration, and frequency are completely different in the new application. In such cases, the bioavailability and safety of the new formulation may be significantly lower in the new therapeutic application, which may even terminate its development [43,44]. Of course, this concern is valid, but we believe that the clinical experience of the first medical use, even in such cases, can be well utilized in optimizing the use of the repositioned active substance for the new indication.

Repositioning is generally classified into three types, based on its starting point: (i) disease-based, (ii) target-based, and iii) drug-based variants [45]. In disease-based repositioning, the most frequently followed version, a new drug candidate suitable for the treatment of a disease is selected from existing drugs. However, an unavoidable question immediately arises: is there any hope that a proven drug with supposedly high selectivity for a certain therapy will also be suitable for other therapeutic purposes? This also leads to another principal question: can the pool of repositioning candidates (approved drugs) be exhausted? Both questions should be considered because, according to the concept that was considered almost an axiom in the 1990s, drugs that are selective only for one target are optimal, regardless of the type of disease.

It may seem surprising that drugs designed for a single target, following the “one drug–one target–one disease” concept, are suitable for repositioning at all. In fact, the reason for this is that most of the secondary successful therapeutic applications of small-molecule drugs are identified by later studies. The successful repositioning stems from the drugs’ polypharmacology (“promiscuity”), which describes the “on/off” target spectrum of a drug, i.e., both the desired and undesired pharmacological effects, of course, from the perspective of a single therapeutic goal.

Polypharmacology in our context means that the effectiveness of these drugs is due to their ability to influence the pathomechanism of the disease through affecting more than one, even several, pharmacologically relevant targets [46,47]. This is also called multi-targeting, which has developed into a successful drug design principle [48,49] and is synonymous with polypharmacology and multi-target pharmacology. It is noteworthy that examples of rationally designed multipurpose molecules have been around for a long time. For example, we ourselves followed this concept when we designed antiarrhythmic drugs with two mechanisms of action by incorporating two pharmacophores into a single molecule [50,51]. Moreover, in 2016, we achieved the multi-target effect in a conceptually new way, which can be considered a special case of indirectly expressed repositioning. We called it the “Metabolism-Activated Multi-Targeting” (MAMUT) approach [52]. In this case, the parent molecule and its active metabolite, itself a known drug, exert synergistic pharmacological effects [50]. The growing importance of the multi-targeting principle is also illustrated by the fact that between 2015 and 2017, 21% of the new molecular entities approved by the FDA were multi-target drugs (31% with combinations), while single-target agents accounted for 34% [53].

In light of the above, polypharmacology can essentially be considered the first pharmacological background and model of disease- and target-based drug repositioning. The great potential of the drug repositioning strategy has now been recognized, and it has become a valuable alternative to the de novo strategy. The wide therapeutic scope of repositioned drugs is particularly impressive, extending to specialized therapeutic areas including rare diseases and neglected tropical diseases [54], as well as unexpectedly and rapidly emerging diseases, such as COVID-19 [55,56]. These areas are significant because they represent difficult-to-satisfy needs and can be particularly risky for de novo drug development. As a result, they are less attractive to investors due to the potential for higher risks and lower profitability.

Meanwhile, a powerful arsenal has been developed to more reliably and quickly identify drug candidates for repositioning. Target- and phenotype-based experimental methods, as well as in silico tools such as virtual high-throughput screening and ligand- and structure-based molecular modelling, have become widely available [45,57,58,59,60,61].

Entering the era of big data, the application and integration of bioinformatics methods has brought particularly significant progress in the selection of candidates for repositioning. In this process, heterogeneous data sets, including omics, pharmacological, and molecular databases, and molecular modelling are selected and integrated [62,63,64,65,66]. Data analysis can then be conducted, usually with the support of artificial intelligence (AI), and can lead to a wealth of information not accessible in traditional ways. Following this theme, with a combination of data science and information technology, various versions are now widely available with expanding scope and increasing efficiency [56,62,67,68,69,70,71,72,73,74,75,76,77]. Importantly, not only can a single drug candidate be selected for repositioning, but drug combinations can also be designed [78].

Recently, complex platforms have been developed for candidate selection, enabling almost discipline-independent application in a user-friendly way for a wide range of pharmaceutical researchers. The Network-based Drug Repurposing and Exploration (NeDRex) platform is an example of this type of new tool [79]. A very interesting computational model, called Drug Repurposing through Exploring Associations using Multi-layer random walk (DREAMwalk), has been published. It is based on a concept described as “a semantic multi-layer guilt-by-association approach that leverages the principle of guilt-by-association—similar genes share similar functions—at the drug-gene-disease level” [80]. Another multi-step computational method, called drug repositioning with attention walking (DRAW), displayed good predictive ability, outperforming known methods [74]. We also refer to a network-assisted approach for the reliable identification of drug–target associations [81]. A deep learning model named MitoReID also performs well [82].

It is also necessary to verify the predictive ability of the repositioning model using (traditional) computational and experimental methods. In an interesting alternative approach, it is not the model but the repositioning project that is qualified [83].

A conceptually different, effective version of the repositioning candidate search approach is based on therapy-relevant data analysis and data mining with bioinformatics tools [84,85]. Clinical and direct therapeutic experience, real-world data (RWD), and real-world evidence (RWE) gained during drug therapy represent a special combination of experimental and data evidence. Additionally, practical experience and observations in the first medical use provide important information about the safety, tolerability, and pharmacokinetic properties of the drug. Systematic efforts have been widely made to exploit the opportunities this evidence provides. With the retrospective analysis of patients’ data stored in electronic health records (EHRs), an unexpected beneficial effect of the drug can be recognized in a therapeutic area different from the original indication [86]. We acknowledge that the full exploitation of this cost-effective approach is limited by non-uniformly organized and defined EHRs, as well as regional variations in legal regulations regarding their access. However, it is clear that these obstacles can be overcome with proper organization and legislation. If this were to happen, EHR data analysis could be implemented as a routine method in the search for repositioning candidates, not only in human medicine but also in the veterinary field.

Overall, we can conclude from the above that there is a rich repertoire of effective repositioning candidate selection approaches available. However, it is striking that until now they have been used almost exclusively in the human field, with almost no examples of their application in the veterinary field.

Returning to the second question posed above: is the existing repertoire of approved drugs large enough to provide suitable candidates for second medical uses to treat a wide variety of diseases? In our opinion, the answer is probably yes. As repositioning candidates, small-molecule drugs can primarily be considered. Although their number shows a somewhat decreasing tendency, we can be optimistic, as their structural and pharmacological diversity represents a long-term relevant and appropriate pool for drug repositioning [73]. Moreover, the collection of potential candidates becomes relatively larger if we take into account drug combinations that contain one or more repositioned drugs as active ingredients [78].

The suitability of the candidate selected for the new therapeutic purpose must be verified for approval. It has been suggested that a carefully selected set of animal models may be appropriate for this purpose [87], but many traditional preclinical animal models have questionable or poor predictive power [88]. We note that a diseased dog model may be particularly suitable for the preclinical qualification of a human drug candidate in the event that the pathomechanism of the dog and the corresponding human disease is similar. Regarding unsuitable, i.e., non-productive animal models, for which a huge number of animals are sacrificed, it is an important development that animal experiments are no longer mandatory for the authorization of clinical trials. The FDA recently declared that new in vitro models (e.g., organoids, organ-on-a-chip) may also be suitable for proving the satisfactory preclinical performance of the drug candidate (see also [88]). This option can thus further accelerate the entire preclinical phase of the repositioning candidate, which can then enter the proof-of-concept clinical study more swiftly. In special cases, depending on the type of treatment and drug formulation, the clinical development of a repositioned candidate may even begin with a phase II study.

### 3.2. Human and Veterinary Drug Repositioning

The path of veterinary de novo drug innovation is usually even shorter, and its risk and cost are lower than those of human de novo drug innovation, as shown by the comparative analysis above. This also applies to the relationship between veterinary drug repositioning and human drug repositioning, as well as veterinary drug repositioning and veterinary de novo innovation. Therefore, we can expect drug repositioning to be a preferred strategy for veterinary drug innovation. Surprisingly, this is not yet the case because, thus far, the primary focus has been on obvious repositioning.

In the common space of human and veterinary drug repositioning, there are four types, depending on the candidate source and direction of repositioning: human–human, human–veterinary, veterinary–veterinary, and veterinary–human. Several representative examples concern the human use of veterinary antiparasitic active substances for the same or similar, i.e., obvious (soft) [89,90,91], but also for non-obvious cancer therapeutic purposes [92]. Human active ingredients are often used in veterinary therapeutic applications with similar, soft indications. According to our definition, these examples are therapeutic extensions rather than repositioning. On the other hand, few examples of non-obvious (hard) repositioning types have been reported, such as antiprotozoal drugs [93]. Their number is further reduced if documented validation is another constraint of this category.

In the drug therapy of companion animals, most examples of drug repositioning are aimed at the treatment of various cancers. With this indication, the repositioning of anti-inflammatory COX-2 inhibitors has long been extensively studied, and some COX-2 inhibitors are now used for anticancer therapy in veterinary practice. Since this area is constantly receiving special attention due to its significant therapeutic potential, it is worth refreshing the rapidly expanding knowledge and outlining new applications. These developments are briefly but systematically reviewed in Section 5.2. In addition to COX-2 inhibitors, other oncology medicines are also being investigated, with representative examples briefly presented in Section 5.1.

The value of both human and veterinary drug repositioning goes far beyond just using a drug in a new therapeutic situation. It can also suggest a new route for de novo drugs and drug combinations in therapeutic areas that are not yet or only partially developed. Nowadays, such a role of repositioning in de novo drug discovery can be very efficiently aided by computational drug design and optimization methods [94]. An example of this is the development of thalidomide analogues. Following the approval of thalidomide for a second therapeutic use for the treatment of leprosy (see above), in an independent repositioning, it was also registered for the treatment of multiple myeloma. This therapeutic application initiated the development of new, more effective, and safer analogues of thalidomide. Thus, lenalidomide and pomalidomide and some combinations have also been approved by the FDA for the treatment of multiple myeloma [95,96].

Last but not least, the possible role of drug repositioning in healthcare should be mentioned. Because of the lower innovation costs, the market price of a repositioned drug can be more favorable than that of a de novo drug. As a result, it can become available to a larger number of patients and, ultimately, reduce the burden on healthcare budgets. This will be discussed further in the following section.

## 4. Business Aspects of Drug Innovation

The attitude and motivation of profit-oriented pharmaceutical companies towards pharmaceutical innovations aimed at human and/or veterinary drug marketing authorization obviously depend on business success. A key factor in this is market exclusivity (market and data exclusivity refer to the length of time an approved medicinal product will be protected from similar competitors; see, e.g., [97]). This is a very important incentive for pharmaceutical innovators, giving them the opportunity to make a profit on their investment [98]. Market exclusivity can be patent-based or “regulatory-based” by a national or regional authority (for basics with real-world examples, see [99]; regarding veterinary medicines, see Exclusivity and Exclusive Marketing Rights Boilerplate for Use in the following documents: Memorandum Recommending Approval, Letter to Applicant, and FOI Summary [100]). In both cases, the exclusivity period begins with the approval of the drug. Business-relevant intellectual property protection issues of drug repositioning are discussed in several publications. We refer to two excellent reviews, the detailed analysis of which is very useful for those interested in more depth [31,44].

For business reasons, the fundamental question is in which way, and for how long, market exclusivity can be ensured. In light of this, it is worthwhile to examine the two modes of market exclusivity: patent-based and regulatory-based exclusivity.

### 4.1. Patent-Based Market Exclusivity

The patent protection period for a new (human or veterinary) medicine is 20 years in most countries. It starts on the date of filing and lasts until the patent expires.

#### 4.1.1. New Chemical Entity

In the case of de novo innovation, the patent is for a new chemical entity (NCE) that has not previously been approved for any medical indication. Such a patent usually provides strong and long-lasting protection for the drug candidate. In practice, however, the duration of market exclusivity is often significantly shorter than the duration of patent protection. This is because, in most cases, the date of the patent application is well before the marketing authorization date of the medicine. The patent application is usually made in the preclinical research (or development) phase and not in the clinical phase. This timing is critical to ensure the originator’s priority, and it is guaranteed, even if a competitor running an overlapping project is preparing a similar patent application. Thus, the patent-based market exclusivity of an NCE does not necessarily prevail over a longer period [99,101]. As a result, national or regional authorities often provide extra innovation incentives to enable the formal extension of a patent protection period. In this way, the market exclusivity can be long enough to achieve an attractive financial result, especially with a new type of product that brings a significant advance, even a breakthrough, in therapy. Interestingly, even this opportunity does not always provide sufficient motivation for de novo innovation because, as described above, the failure rate is extremely high and so the financial risks are greater. According to a recent study, this is why companies frequently prefer less expensive and less risky incremental “pseudo-innovative” or “evergreen” strategies [102].

#### 4.1.2. Second Medical Use

In many countries and regions, a second medical use patent is possible. The claim formats of patents for second medical use vary depending on the patent authority [103,104,105]. According to the European Patent Office (EPO), the second medical use of a drug is also patentable [106]. Consequently, the second medical use can gain market exclusivity with patent protection, which lasts 20 years in both the USA and Europe. However, the Asian region is different. Such protection is not uniformly allowed in certain countries, and the second medical indication cannot be patented [107].

The benefits of patenting a second medical use also depend on the patent status of the active substance. There are two scenarios. In the first, the active substance is still under the patent protection of the inventor (the originator of the first patent); repositioning is only practical for the originator to avoid the interdependence of the patent for the NCE with the second use patent of two different patentees or suchlike. In the second scenario, the active substance is already generic. In this case, in our opinion, the second medical use is worth patenting. However, the chances of patenting the second medical use for a generic drug appear to be modest [73,108]. This is because fulfilling the novelty and “inventive step” criteria requires very strong evidence. As a consequence, the repositioning of generic active substances to a second medical use can be unattractive in terms of commercial success. Fortunately, this conclusion is hardly valid in all cases, as recent comprehensive expert studies based on data from the EPO have confirmed. Significantly, the studies found that “European Patent Office data show an increasing number of patents for new medical uses of known products” and “Obtaining European patent protection for medical uses of known products is not a key factor limiting repurposing activity” [109,110]. It is also important to mention that a similar, broader analysis also documents that there is a real chance of patenting a new use of an earlier invention [111].

The patentability of veterinary medicinal products is similar to the patentability of human medicines in many respects (here we also refer to the FDA definition of generic veterinary drugs [112]) but does not lack veterinary-specific issues. For example, cross-species patenting can be especially challenging. The approval of a patent application with such claims requires a very thorough examination of prior work for novelty and strong arguments for non-obviousness (for details, see [113]).

### 4.2. Regulatory-Based Market Exclusivity

Regulatory-based market exclusivity (in short, regulatory exclusivity) periods for each human and analogue veterinary category are defined in relevant EMA and FDA documents; see [114,115] and references therein.

The regulatory exclusivity periods for a human NCE drug, an orphan drug, and a new clinical investigation are different. For an NCE, the exclusivity period, which blocks the approval of generics, is usually much shorter than patent-based exclusivity and varies by region and by type of drug. It is 5 years in the USA and 10 years in the EU. For an NCE drug used in dogs, it is 10 years in the EU.

The new clinical investigation category relates to the second medical use, thus also drug repositioning. A brand-name human drug with an active ingredient that has been approved before may be awarded a three-year exclusivity if a different disease or condition the drug can treat is identified. To gain this approval, new clinical studies are required in humans. In the case of veterinary medicinal products, in special cases, it can be 5 years.

There is also the orphan drug category for the treatment of rare human diseases, which also has significant repositioning relevance. The contribution of orphan drugs to the drug portfolio is increasing. In the case of medicines to treat rare human disease, regulatory exclusivity lasts longer (7 years in the USA) than for an NCE because the incentives motivating profit-oriented companies are modest due to the limited number of patients. An orphan medicine is a drug which is used to treat a rare human disease (defined as affecting in the USA <200,000; in Europe less than 5 in 10,000 persons [116]) and which receives governmental support for development. The estimated number of such diseases is around 7000–8000, but treatment is only available for a few hundred [117]. The strong motivating effect of supportive regulation on orphan drug innovation is evidenced by the growing number of approved orphan drugs; in 2023, for instance, 28 human orphan drugs (out of 55 small-molecule drugs, which corresponds to 51%) were approved by the FDA. The increasing role of drug repositioning in this area is also important [118].

In the veterinary area, in special cases, a veterinary medicine can be authorized in the Minor Use/Minor Species (MUMS) category in USA, which may also provide longer market exclusivity [119]. The role of a drug repositioning strategy in the development of human orphan drugs may have certain limitations, as discussed in excellent publications [36,120]. Nevertheless, the situation of a drug repositioning strategy in this area is changing in a favorable direction. In this regard, we mention that appropriate databases and data analysis platforms are emerging, while incentives are being introduced to encourage various stakeholders to join and actively participate in the orphan drug repositioning program [121].

From the market exclusivity analysis for the second medicinal use, we can conclude that patenting the repositioned drug may be feasible and provide long-term multi-country exclusivity. Moreover, in the field of special diseases, such as rare diseases, an attractive regulatory-based exclusivity period can be obtained in the EU and the USA. In light of this, it seems a bit superficial that the uncertain market perspective is often cited as the main limiting factor for all variants of drug repositioning.

It must be admitted that such a skeptical opinion is not completely unfounded. In particular, off-label use can reduce the market results of drug repositioning. Therefore, it is in the legitimate interest of the interested party to minimize the limiting effect of off-label use on the commercial success of the repositioning. We present two examples of such solutions in Section 4.3.

### 4.3. The Role of Off-Label Use in Drug Repositioning

The off-label (i.e., outside of the labeled) and cross-label (a subtype of off-label) use of a drug means that medical doctors may use a drug for purposes other than those approved by the FDA or the EMA. (For example, the “DrugCentral 2023” database contains data on off-label human drug use [122].) If the formulation of the first and second use is the same, it can limit the commercial success of the patented second medical use.

The FDA also allows extra-label or off-label use in the veterinary area [123]. Accordingly, under certain circumstances, veterinarians may use an approved human or animal drug for a therapeutic purpose that is not listed on the drug’s label. In the EU, using human-authorized medicines in animals is only allowed according to a specific drug cascade, which is a risk-based decision tree [124]. When there is no suitable veterinary medicine authorized in a territory for a specific condition, veterinarians are permitted to treat animals under their care according to this cascade, to avoid unacceptable suffering. Prescribing decisions in accordance with the cascade should be made on a case-by-case basis [124].

The business-limiting effect of off-label use can be suppressed or even eliminated by strict regulatory control and the independent approval of medicines containing the same active ingredient for different indications. The repositioning of sildenafil is a good example. Viagra and Revatio, approved by the FDA in 1998 [125] and 2005 [126], respectively, contain the same active ingredient, sildenafil. Viagra is approved for the treatment of erectile dysfunction, while Revatio is for the treatment of pulmonary arterial hypertension (PAH). Their dosage forms and schedules are also different. Thus, the use of one drug for the other therapy cannot be realized either legally or practically.

A recent example of the same active substance marketed for different indications under different brand names is semaglutide, a glucagon-like peptide 1 (GLP-1) receptor agonist. The FDA approved Ozempic for diabetes in 2017 and Wegovy for weight reduction in 2021. In 2024, the FDA extended the indication for Wegovy to include the risk of serious heart problems, especially in obese or overweight adults [127]. With all this, the business success has been outstanding, and the company has seen record profits—Novo Nordisk has achieved the largest market capitalization in Europe [128]. The story of semaglutide is not over yet. The horizon of GLP-1 agonists may expand further due to their possible atherosclerosis-reducing effect [129].

The above analysis confirms that the business potential of drug repositioning can be significant with a strategy that takes into account all possible constraints (e.g., patent vs. regulatory exclusivity) and supporting conditions (e.g., incentives).

## 5. Drug Repositioning for Oncological Diseases

### 5.1. Current Aspects of Drug Therapy in Oncology

Human cancer is a heterogeneous disease with more than 200 variants [130]. According to a report by the International Agency for Research on Cancer (IARC) [131], cancer caused 20 million new cases and 9.7 million deaths worldwide in 2022 alone. Cancer cases are expected to increase to 35 million by 2050—a 77% increase from 2022 [132]. The cumulative costs of cancer over the next two decades are estimated at 1300 billion [131].

Cancer is also one of the most common diseases in companion animals. The incidence is particularly high in dogs, with 45% of dogs older than 10 years suffering from cancer. The death rate of companion animals with cancer is remarkably high [133].

Of course, there is still much to be done to achieve a significantly more favorable scene in human and companion animal oncology. There is a common opinion that comprehensive cancer prevention can fundamentally change this unfavorable position. Prevention programs and preventative measures are much more developed for cancer in humans, although their practical application still falls short of what is necessary [134]. According to a recent analysis, up to 1.3 million human cancers could be prevented if the prevention policies of the best performing countries were followed in Europe [131]. However, it is unlikely that we will arrive at this destination in the near future. The approach to this goal is still slower than it should be, mainly because the implementation of the concept and its guidelines is not progressing fast enough.

An effective therapeutic arsenal is needed to stop or at least slow down the progression of the disease, inhibit the transformation of cancer into more aggressive, untreatable forms, and improve patients’ quality of life with relevant interventions. This demand exists both for humans and companion animals with cancer. Epidemiological data show that the trends in oncological diseases in humans and companion animals are roughly similar. This is due to common contributing factors in humans and dogs, such as increasing average age, similar adverse environmental effects, and the similar pathophysiology of cancer in many respects. This similarity is especially close between natural dog cancers and the corresponding human diseases.

This has two important practical implications: one related to human drug innovation and the other to both human and dog cancer pharmacotherapy. The pathomechanism of cancers in dogs (and other companion animals), as well as the effectiveness of their drug therapy, can serve as a model for human drug research and development. This model is much more reliable than traditional preclinical rodent models. As a result, the failure rate of human drug innovation is likely to be much lower. In addition, the advantages of the repositioning strategy can prevail even more. Clinical experience with new human anticancer drugs developed in this way can then be used to optimize anticancer therapy in dogs, and its results can then be used again in human drug innovation and therapy [135]. Then, the cycle can begin again.

The pharmacological basis of this strategy is broadly consistent with the recently established and rapidly evolving field of comparative oncology [136], including a comparative analysis of biological aspects (see, e.g., [137]). In addition, this approach also motivates the development of a common platform for human and dog oncology drug innovation and drug therapy on a rational basis. In this context, we note that it is worthwhile to jointly analyze human and veterinary drug innovation, including repositioning, and utilize their results in both areas (we will return to this proposal in the sixth and seventh sections). In essence, this strategy means the extension of the One Health concept to drug repositioning.

Oncology therapy requires both diagnostics and effective drugs. Diagnostic tools include all types of biomarkers [138], which are preferably used in combination [139,140] for precision medicine. Treatments require a drug portfolio enriched with highly effective new drugs and drug combinations for traditional chemotherapy and breakthrough targeted and immunotherapies. In the last ten years, favorable development has taken place in all these areas of human oncology. The most important results include improved therapeutic success rates measured by patient-centered outcomes [141] in several previously untreatable areas of human oncology, as well as a continuous overall 29% decrease in the death rate of human cancers between 1991 and 2020. Of course, this significant development also has cost implications. The expanding therapeutic repertoire and new therapies, especially biological therapy, come at a high cost. Primarily, the increasing expansion and the development of additional new drugs are responsible for the significant growth in the global human oncology drug therapy market from USD 135.8 billion in 2022 to a projected USD 448.6 billion in 2032 [142].

Due to fiscal-based health policies, the rapidly increasing costs that stretch the financial limits of the healthcare sector can hinder the widespread adoption of new high-cost therapies. Of course, it is an obvious and legitimate demand on the healthcare system to prove the significant therapeutic benefits. At the same time, a general restrictive policy is of dubious value because it does not take into account the favorable consequences of more effective therapy for both patients and society (e.g., patients’ extended lifespan, improved quality of life, and ability to work for a longer period). However, the cost of medicine is a significant expense. This can be reduced by improving the performance of drug innovation, for example, with more reliable, cheaper, and shorter drug repositioning compared to de novo innovation.

The development of oncological therapy for companion animals is undoubtedly advancing but still in much smaller steps than human therapy. The market segments, with their increasing value and size, reflect this, although absolute figures fall far short of human domains. (PET Cancer Therapeutics Global Market Size 2022: USD 369 million, 2032: USD 1 bn [143], according to a significantly different estimation, the Global Veterinary Oncology market size 2022: USD 260 million, 2033: USD 800 million [133]). One reason for the slower development is that the extension of human medicines to the veterinary field is still far from being fully exploited. The Drug Central database, presenting and commenting on human vs. veterinary datasets, also confirms this [144,145].

This trend can be recognized in the veterinary field of recently introduced anticancer and immunotherapy drugs. Such highly effective drugs, already successfully used in human oncology practice, are very modestly represented in the spectrum of veterinary anticancer drugs. For example, significantly fewer monoclonal antibody (mAb) type agents are available in anticancer therapy for dogs than in human therapy, and immunotherapeutic agents are also lagging behind [146]. These facts are quite surprising, since the driving force behind the complete characterization of canine cancers is not only the need for effective therapy but also their use as human models (see above). The molecular depth description of the tumors of dogs (and cats), the understanding of their oncological diseases based on omics, pharmacogenetics and genomics, and oncological systems pharmacology, as well as precision diagnostic methods, still lags behind the knowledge in human areas. Moreover, limited resources are not always used correctly. It is no wonder that the essentially schematic and insufficiently justified application of human anticancer therapies to dogs often fails.

In current veterinary oncology practice, chemotherapy is responsible for the majority of treatments, followed by targeted therapy and immunotherapy drugs. The contribution of the latter two is less significant than in human therapy. Drug combination therapy accounts for only a small part of the entire veterinary oncological pharmaceutics portfolio. Chemotherapy treatments are carried out in two ways. The conventional version is used intermittently, at the highest tolerated dose, primarily to combat rapidly progressing and metastatic malignant tumors. Metronomic chemotherapy aims at inhibiting angiogenesis and is used in low oral doses at shorter intervals. Thus, the toxic effect on normal cells is minimal, and the therapy can be conducted outside of hospital. Both types of chemotherapy are available in veterinary practice [147].

Both in human and veterinary anticancer therapy, the therapeutic effectiveness of a chemotherapeutic agent can be significantly reduced or even completely prevented by resistance to anticancer drug therapy. Resistance affects virtually any type of anticancer treatment. It may be present at the beginning of therapy or may develop during the treatment. In order to optimize anticancer therapy, the possibly multi-component mechanism of resistance should be explored [148,149,150,151,152].

Generally, only one factor is studied; in most cases, it is the membrane transporter P-glycoprotein (permeability glycoprotein or multidrug resistance protein 1; a member of the ATP-Binding Cassette transporter superfamily; it is also called ABCB1). Indeed, the P-glycoprotein transporter is the main player in the most common resistance mechanism of both veterinary and human anticancer pharmacotherapy. It is overexpressed in tumor cells of different cancer diseases and can remove a wide range of structurally diverse active substances from tumor cells, thereby reducing the effectiveness of the therapy [150,153]. In such cases, the inhibition of P-glycoprotein (or the corresponding efflux transporter) seems to be a reasonable intervention. Nevertheless, this approach has several limitations. The disease- and site-selective blockade of P-glycoprotein is difficult to achieve in practice, while a non-selective blockade, also affecting normal cells, can cause toxic effects [153]. Furthermore, transporter inhibition can also be harmful by contributing to serious drug–drug interactions [154]. P-glycoprotein transporter and cytochrome P450 (CYP) enzymes, participating in drug metabolism, may have common substrates, depending on the species. This can also result in different substrate behavior in humans and dogs with different therapeutic consequences. In addition, the efflux mechanism of the transporter can also be influenced by host genetic factors expressing different P-glycoprotein polymorphs. For example, mutations in MDR1 can result in non-functional P-glycoprotein. Such a case has been recognized in dog breeds, with severe toxic consequences [155]. When treating such dogs, the dose of anticancer drugs that are P-glycoprotein substrates should be reduced [156]. Notably, the same also applies to human patients with the respective mutation.

The list of incomplete, dubious, and sometimes misleading studies on drug resistance is surprisingly long. We refer here to a rigorous critical review published in 2018, specifically regarding therapy in dogs. The review recommends genetic testing in dogs, which we wholeheartedly agree with. However, genetic testing should be conducted with caution: “In the United States alone, some 70% of households own pets. Done right, the use of genetic testing in companion animals could be a powerful way to better connect people to the possibilities of genetics for treating disease. Done wrong, it could erode trust in science for an increasingly skeptical public” [157].

Perhaps, at least in part, because of the critical comments of such publications, meaningful genetic analyses have been reported in the last 3–4 years. For example, a real-world study reported in 2023 described the survival outcomes of 2119 dogs with tumors, and in a subset of 1108 dogs, genomics for mutations were analyzed and therapy-relevant mutations were identified [158]. In another excellent study related to the precision anticancer therapy of dogs, cancer-relevant mutations were identified by genomic analysis. Their diagnostic and therapeutic value in clinical practice was also demonstrated [159].

The accelerating spread of this type of investigation and its extension to all omics may encourage the desired renewal of veterinary anticancer strategies. One of the important weapons in this arsenal is combination treatments. A comprehensive review analyzing the oncological applications of drug repositioning, including combinations, and discussing its advantages and difficulties appeared very recently. It also covers aspects independent of the therapeutic area (e.g., IP) [31].

The majority of opinions—including ours—agree that there is a place for drug combinations (there are fixed-dose combinations and combinative treatments; these terms are also used in an overlapping meaning; we uniformly use “combination” for both versions) in the anticancer pharmacotherapy portfolio [31,160,161,162]. Although the mechanism- and omics-supported design of drug combinations is now possible in many oncological disease areas, there is still no model available to predict (not only qualitatively but also quantitatively) the complete therapeutic profile of the combination based on its components. The way in which such treatments are developed has generally been empirical. Many of them were based on real-world data analysis or simply clinical observations and, occasionally, intuitive thoughts and holistic, network analysis. Often, one of the components is selected from non-oncology therapeutic areas, i.e., repositioned to an anticancer combination; nevertheless, this practice may have advantages, but it also has its own theoretical and practical limitations.

Recently, a few publications have described examples of drug repositioning for the anticancer therapy of companion animals. We refer to the following articles and reviews for illustrative purposes. An instructive comprehensive review of repositioned drugs that can be used in oncology therapy for dogs and cats appeared in 2023 [163]. Another interesting review describes the treatment options for dog mammary carcinoma, including chemotherapy, in which human anticancer and repositioned (COX-2 inhibitors) agents are used [164]. Finally, we refer to Lapatinib, a kinase inhibitor used to treat human breast cancer, whose repositioning is currently being investigated for the treatment of dog urothelial carcinoma [165].

In the next part, this section will systematically review the repositioning of COX-2 inhibitors, a frequently investigated and expanding drug family in the treatment of veterinary oncology. It also illustrates the utility of the common space of human and veterinary drugs in selecting candidates for repositioning.

### 5.2. Drug Repositioning of COX-2 Inhibitors for the Treatment of Dog Cancers

Cyclooxygenases play an important role in the synthesis of prostaglandins. Arachidonic acid, a fatty acid with 20 carbonic atoms and an essential component of eucaryotic cell membranes, is the precursor of eicosanoids, produced by an inflammatory pathway initiated by Phospholipase A2. The cyclooxygenase pathway, which generates prostaglandins (PGD2, PGE2, PGF2α, PGI2) and thromboxane (TxA2) via COX-1 and COX-2 enzymes [166] is one way for the formation of eicosanoids. The COX-1 isoenzyme is constitutively produced in several cells and tissues, while COX-2 isoenzyme production is inducible, in which inflammatory mediators, cytokines, and growth factors play an important role [167,168]. Nonsteroidal anti-inflammatory drugs (NSAIDs) reduce inflammation and relieve pain, such as in rheumatoid arthritis and osteoarthritis, by inhibiting COX enzymes. COX-2 selective agents have been widely used in human and companion animal medicine to treat these conditions. In fact, the long-term inhibition of the COX-1 isoenzyme can lead to a number of side effects due to the loss of the protective function of eicosanoids. These can include renal failure or gastrointestinal ulcers [169]. However, the COX-2 isoenzyme also plays a role in the protection of these organs [170,171]. In addition, compounds that are specifically selective for COX-2 can greatly increase the incidence of thrombotic events, as the inhibition of PGI2 will lead to an imbalance between prostacyclins and TxA2 and therefore to increased platelet aggregation. Recognizing this effect, several coxib compounds (e.g., valdecoxib) have been withdrawn from the market, with celecoxib remaining the dominant substance available.

NSAIDs used in companion animal medicine were first adopted by veterinarians from human medicine. Metamizole sodium was authorized in veterinary medicine in the 1950s, followed later by more COX-2-selective agents such as carprofen or meloxicam. These latter agents are still widely used today to treat inflammatory conditions in dogs and cats and to reduce pain or fever. However, the average age of animals has been extended, which has led to an increased incidence of osteoarthritis, particularly in dogs [172], and an increased need for professional veterinary care. However, to treat this condition, a need emerged for COX-2-selective agents that could be safely used in animals in the long term. Rather than veterinary medicine adopting the coxibs used in human medicine, a class of coxibs used exclusively in veterinary medicine began to be developed.

The first veterinary coxib for dogs to be centrally authorized by the EMA was firocoxib in 2004 [173]. This compound was followed by a number of others, such as mavacoxib [174], robenacoxib [175], cimicoxib [176], deracoxib [177], and enflicoxib [178], which was approved by the EMA in 2021. Deracoxib has only FDA approval, while all the other veterinary coxibs are also centrally approved by the EMA and widely used across the EU. Of the coxibs listed above, robenacoxib is the only one that is approved for cats and is indicated for short-term pain relief following soft tissue surgery in addition to chronic orthopedic pain. There is also a significant difference between the authorized veterinary coxibs regarding their half-lives. Most of the substances should be administered daily, but for long-term use, enflicoxib should be administered weekly (t_1/2_ 20 h) and mavacoxib monthly (t_1/2_ 17 days). The NSAIDs authorized in companion animal medicine are summarized in Table 1 in the order of the date of authorization, with their respective target species, indication, and route of administration. In Table 2, the IC_50_ values and COX-1:COX-2 ratios for each NSAID are summarized.

In recent years, the use of NSAIDs in cancer therapy has also become more prominent. Botha et al. first demonstrated the involvement of PGE2 and PGF2α in human esophageal carcinoma cells in 1986. Prostaglandins reduced tumor invasion and metastatic ability in nude mice [179]. Since then, a large amount of evidence has supported the role of prostaglandins in tumor development, angiogenesis, and metastasis in a variety of tumor types [167,180]. Therefore, COX inhibitors can be used to induce apoptosis and arrest tumor cell division [181].

Although the use of celecoxib alone has been successful in the treatment of hepatocellular carcinoma in humans, myeloid leukaemia-1, and breast cancer [182,183] and can reduce multidrug resistance (MDR) by inhibiting P-glycoprotein [184,185], COX inhibitors should be seen primarily as a combination partner in chemotherapy. In the case of breast cancer, it has previously been demonstrated that COX-2 inhibition can reduce metastasis due to the inhibition of metalloproteinase 1 (MMP1) enzymes [186]. Although the efficacy of COX-2-selective compounds has been mainly investigated in tumor therapy, there is also growing evidence that COX-1 plays a key role in certain tumor types [187]. In the following, we will systematically review the available publications in the literature that have investigated the antitumor efficacy of NSAIDs authorized in companion animals.

Pubmed and Web of Science were used to search for publications. The search terms were (“cyclooxygenase 1” OR “cyclooxygenase 2”) AND ((“dogs”) AND (“cancer” OR “tumour”) AND (“Nonsteroidal Anti-inflammatory Drug” OR NSAID)). The search was performed over the last ten years (2014–2024), with the time of the query being 21 March 2024. The search resulted in seventeen hits on Pubmed and eight hits on Web of Science. From these, we filtered out irrelevant articles and overlaps, resulting in a total of 11 publications.

**Table 1:** NSAIDs authorized in dogs and cats and the product name of the first original product, in the order of the date of authorization, with their respective target species, indication, and route of administration, based on EMA [188], FDA [189], and national data [190]. National authorization date indicates the date of the first authorization in Hungary.

Active Ingredient INN	Authorized Veterinary Medicinal Product	Registration Number of the Product	Authorization Date	Type of Authorization	Species	Indication	Administration Route
Metamizole sodium ^H^	Algopyrin	3915/1/17 NÉBIH ÁTI	1 January 1956	National authorization	Dog	Fever management, relief of abdominal pain symptoms, relief of esophageal spasm in the case of esophageal obstruction, muscle and joint pain, and post-operative pain relief.	IM
Phenylbutazone ^H^	Phen-Pred	2284/1/07 MgSzH ÁTI	8 November 1999	National authorization	Dog	For the treatment of inflammatory diseases of the musculoskeletal system (arthritis, tendinitis, rheumatism), hyperthermia, heat stroke, and inflammatory processes of traumatic or bacterial origin.	IV, IM, PO
Tolfenamic acid ^H^	Tolfedine	2084/1/06 ÁOGYTI	23 May 2000	National authorization	Dog	Treatment of painful bone, joint, and musculoskeletal disorders.	IM, SC, PO
					Cat	For the treatment of fever, NSAIDs can be used as an additional treatment.	SC, PO
Carprofen ^H^	Rimadyl	2617/1/09 MgSzH ÁTI	13 November 2002	National authorization	Dog	Relieving inflammation and pain caused by musculoskeletal pathologies and degenerative joint diseases and post-operative pain relief.	IV, IM, PO
					Cat	Post-operative pain relief.	
Ketoprofen ^H^	Ketofen	2211/1/07 MgSzH ÁTI	11 June 2007	National authorization	Dog	For the treatment of acute, painful, inflammatory lesions of the bones, joints and musculoskeletal system, especially in cases of arthrosis, trauma, dislocation, disc herniation, and oedema. For the treatment of fever and pain after surgery. Treatment of chronic pain due to osteoarthritis or musculoskeletal disorders.	PO
					Cat	For the treatment of acute, painful, inflammatory lesions of the bones, joints, and musculoskeletal system, especially in cases of arthrosis, trauma, dislocation, disc herniation, and oedema. For the treatment of fever and pain after surgery.	
Meloxicam ^H^	Metacam	EMEA/V/C/ 000033	7 January 1998	Central authorization, EMA	Dog	Reduction in inflammation and pain relief in acute and chronic musculoskeletal disorders. Reducing post-operative pain and inflammation following orthopedic and soft tissue surgery.	IV, SC, PO
					Cat	Post-operative pain relief after hysterectomy, ovariectomy, and minor soft tissue surgery.	
* Tepoxalin ^V^	Zubrin	EMEA/V/C/ 000057	13 March 2001	Central authorization, EMA	Dog	Used to reduce pain and inflammation (soreness) due to osteoarthritis.	PO
Nimesulide ^H^	Zolan	2869/1/11 MgSzH ÁTI	23 April 2004	National authorization	Dog	To reduce inflammation and relieve bone, joint, and muscle pain.	PO
Firocoxib ^V^	Previcox	EMEA/V/C/ 000082	13 September 2004	Central authorization, EMA	Dog	To reduce inflammation and pain during osteoarthritis. To relieve pain and inflammation caused by soft tissue, orthopedic, and dental surgery.	PO
Mavacoxib ^V^	Trocoxil	EMEA/V/C/ 000132	9 September 2008	Central authorization, EMA	Dog	For the treatment of pain and inflammation associated with degenerative joint disease, if the duration of the proposed treatment exceeds one month.	PO
Robenacoxib ^V^	Onsior	EMEA/V/C/ 000127	16 December 2008	Central authorization, EMA	Dog	Treatment of pain and inflammation associated with chronic osteoarthritis. Treatment of pain and inflammation associated with soft tissue surgery.	SC, PO
					Cat	Treatment of acute or chronic pain and inflammation associated with musculoskeletal disorders. To reduce moderate pain and inflammation associated with orthopedic surgery.	
Cimicoxib ^V^	Cimalgex	EMEA/V/C/ 000162	18 February 2011	Central authorization, EMA	Dog	For the treatment of pain and inflammation associated with osteoarthritis and pain related to orthopedic or soft tissue surgery.	PO
Deracoxib ^V^	Deramaxx	807-307-6	11 January 2015	FDA authorization	Dog	The control of pain and inflammation associated with osteoarthritis, orthopedic surgery, or dental surgery.	PO
Enflicoxib ^V^	Daxocox	EMEA/V/C/ 005354	20 April 2021	Central authorization, EMA	Dog	Treat pain and inflammation associated with osteoarthritis (or degenerative joint disease).	PO

* Tepoxalin was withdrawn by the EMA in 2012 but remains on the market in the USA under FDA approval. H: Active substance taken from human medicine; V: active substance authorized only in veterinary medicine. IM: intramuscular; IV: intravenous; PO: per os; SC: subcutaneous.

**Table 2:** IC_50_ values and COX-1:COX-2 ratios for NSAIDs used in companion animals. The values shown in the table are the extreme values found in the literature.

Active Ingredient INN	Species	IC_50_ − COX-1 (µM)	IC_50_ − COX-2 (µM)	COX-1:COX-2	Reference
Metamizole sodium	Dog	350	>1000	<1	[191,192]
Phenylbutazone	Dog	17	28	0.6–9.7	[193,194,195]
Tolfenamic acid	Dog	-	3.53	0.06–15	[196,197,198,199]
	Cat	-	-	-	
Carprofen	Dog	4.4–380	1–161	2.4–129	[193,195,197,199,200,201,202,203]
	Cat	8.9–26.6	0.9–1.6	5.5–28.1	[195,202,204]
Ketoprofen	Dog	0.1–4.9	0.1–13.5	0.36–0.8	[193,195,197,199,203]
	Cat	0.02–0.5	0.5–48.5	0.009–0.05	[205,206,207]
Meloxicam	Dog	1–23.7	0.1–1.9	7.2–12.3	[193,195,197,199,203]
	Cat	1.35–4	0.5–1.2	2.7–3.5	[204,205]
* Tepoxalin	Horse	0.04	0.06	0.35	[208]
Nimesulide	Dog	6.4–20.3	0.17–1.6	13–36.3	[203,209]
Firocoxib	Dog	56–119.1	0.16–0.31	380–384	[200,210]
Mavacoxib	Dog	8.7	0.4	22.2	[211,212]
Robenacoxib	Dog	7.8–10.8	0.04–0.07	128.8–141.3	[203,213,214,215]
	Cat	4.5–28.9	0.04–0.12	32.2–502.3	[203,205,206,213,214,216]
Cimicoxib	Dog	-	-	-	-
Deracoxib	Dog	4.9–9.9	0.2–0.4	12.0–61.5	[194,200,203]
Enflicoxib	Dog	37.5–334	2.9–11.7	3.2–113.9	[217,218,219]

* Only data from horses are available for tepoxaline.

Pang et al. [220,221] isolated cancer stem cells (CSCs) from a primary patient with canine osteosarcoma. These CSCs exhibited resistance to conventional chemotherapy and showed increased COX-2 expression compared to parental cells. Despite increased COX-2 levels, inhibiting COX-2 did not affect CSC growth or chemotherapy resistance but did prevent sphere formation in daughter cells. Similar effects were observed in human osteosarcoma cell lines. These findings suggest that while COX-2 inhibitors do not impact CSC viability directly, they do influence the formation and maintenance of cancer cells, indicating a potential therapeutic role for COX-2 inhibitors in osteosarcoma treatment. The study also highlights dogs as a potential preclinical model for human osteosarcoma therapeutics, given the similarities observed between canine and human cells.

Similarly, Seo et al. [222] examined the effects of celecoxib on canine melanoma cell lines, finding it induced cell cycle arrest and apoptosis, especially in cells with high COX-2 expression. They found that celecoxib caused G0/G1 phase arrest and activated caspase-3. These results indicate that celecoxib might be effective as a chemotherapeutic agent against canine malignant melanoma.

In another study, Rathore et al. [223] explored piroxicam’s effects on oral squamous cell carcinoma (OSCC) in humans, dogs, and cats. Co-treatment with piroxicam and AB1010 significantly inhibited OSCC cell proliferation, reducing COX-2 expression by inhibiting nuclear factor-kB and AP-1 transcription factors.

Saito et al. [224] investigated COX-2 inhibitors (meloxicam, etodolac, celecoxib) in canine mammary tumor cells. All inhibitors suppressed cell growth, and etodolac and celecoxib were particularly effective in inducing apoptosis via the mitochondrial pathway. Celecoxib was highlighted as a promising therapeutic candidate, suggesting COX-2 inhibition as a new strategy for treating canine mammary tumors.

Vahidi et al. [225] found that combining citrate with celecoxib had a synergistic antitumor effect against canine mammary gland tumor cells, enhancing apoptosis and inhibiting cell proliferation more effectively than either compound alone.

Arenas et al. [226] evaluated firocoxib as adjuvant therapy for highly malignant canine mammary tumors (HM-CMTs), finding it significantly improved disease-free survival (DFS) and overall survival (OS) compared to controls, supporting its use in treating HM-CMTs.

Yoshitake et al. [227] examined the antitumor effects of NSAIDs on canine cancer cell lines, finding no strong correlation between COX expression and NSAID sensitivity. They identified several genes potentially involved in NSAIDs’ antitumor effects, suggesting COX/PG-independent pathways.

Hurst et al. [228] explored the effects of mavacoxib, a COX-2 inhibitor, on various canine and human cancer cell lines. Mavacoxib was found to induce apoptosis and inhibit cell migration in a COX-2-independent manner. This study suggests mavacoxib as a promising cancer therapeutic, capable of reducing cell viability and migration across multiple cancer types regardless of COX-2 expression levels.

Alonso-Miguel et al. [229] assessed the efficacy of a firocoxib in combination with toceranib phosphate and oral cyclophosphamide (multidrug therapy) compared to COX-2 inhibitor alone in dogs with inflammatory mammary cancer (IMC). The combination therapy significantly improved the overall survival time and disease-free survival compared to single-drug therapy, suggesting the potential benefits of using COX-2 inhibitors in a multidrug regimen for treating highly malignant IMC.

Brandi et al. [230] evaluated the pro-apoptotic effects of the COX-2 inhibitor firocoxib in canine mammary tumor (CMT) cell lines and in vivo. The study found that firocoxib induced apoptosis in CMT cells and increased COX-2-positive apoptotic cells in treated dogs. These results propose firocoxib as a potential neoadjuvant treatment for canine mammary cancer, showing efficacy both in vitro and in vivo.

These studies collectively underscore the potential of COX-2 inhibitors as therapeutic agents in various canine cancers, including osteosarcoma, melanoma, oral squamous cell carcinoma, and mammary tumors. The findings support the use of COX-2 inhibitors, both as single agents and in combination therapies, to inhibit tumor growth, induce apoptosis, and potentially improve survival outcomes in cancer treatment. The similarities between canine and human cancers also highlight the utility of canine models for preclinical cancer research, offering insights that could inform human cancer therapies.

## 6. One Health Approach to Drug Repositioning

The One Health concept includes human and veterinary medicine (for a concise description, see [231]). In both fields, zoonotic diseases [232], antimicrobial resistance [233], and emerging infectious diseases [234] are of primary interest due to their increasing global importance. In the last decade, the importance of the One Health concept has been recognized, interpreting healthcare in a wider scope and connecting comparative and translational medicine [233,235]. It is easy to see that this relationship essentially means that the One Health concept connects human and animal drug development, from which it can be deduced that companion animals, especially dog models, can play an important role in the development of human drugs, e.g., vaccines and oncology preparations [233].

Our interest in the One Health concept is in the connections between human and veterinary drug repositioning. It is noteworthy that, traditionally, the two repositioning pathways are separate, although their main phases and many components may be similar or even overlap. Thus, it seems reasonable to us that human and veterinary, especially dog, repositioning pathways may converge to a common pathway. Due to this convergence, many tools of drug repositioning can be used together for human and veterinary drug development. In the preclinical phase, the first part is the selection of candidates for in vivo testing by screening known drugs using in silico and in vitro (possibly, 3D cell cultures and organ-on-a-chip) methods. In the second part, in vivo experiments are performed, especially in diseased dog models, to provide sufficient evidence for human or animal clinical trials. Thus, in a favorable case, the approach can also lead to drugs in the fields of human and dog therapy.

We are building a repositioning platform to put our vision into practice. This platform, which we call the One Health Drug Repositioning Platform, brings together all the important participants and tools needed for both veterinary and human repositioning and their joint management.

The actions have two key elements. The function of the first element is strategic, opinion-forming, and coordinating. This involves the creation of a forum for all relevant stakeholders in order to exchange ideas and make strategic decisions. This should include experts from academia and pharmaceutical companies and clinicians active in human and veterinary drug research, drug development, and clinical practice, as well as intellectual property experts and innovation policy makers. The second element contains the collection of relevant tools. This should include in silico, in vitro, preferably 3D cell and organ-on-a-chip models, and, if necessary, in vivo, preferably diseased dog, models. In silico methods are data analysis methods, such as AI, which are suitable for analyses of large and heterogeneous databases, including human and dog omics, disease networks, pathomechanism-relevant data, real-world clinical data, data from publications (text mining), and drug-structure- and drug-therapy-relevant data. All or part of these data are analyzed comprehensively and comparatively, primarily to identify associations and relationships, with the ultimate aim of identifying repositioning candidates for both human and veterinary drug repositioning.

Overall, it can be concluded that coordinated human and veterinary drug repositioning according to our approach can be shorter and more effective than the traditional approaches via separate routes. Thus, the one-route approach is worthy of consideration. Last but not least, we note that our approach supports the effort to reduce the number of animal experiments, especially those of dubious value and often requiring the sacrifice of many animals.

## 7. Conclusions and Perspective

Interest in drug repositioning has been increasing over the past few years, and this strategy has become a worthwhile alternative and complement to de novo drug innovation. The more favorable position of drug repositioning is primarily due to two factors. The first concerns the repositioning methodology. Since the early 2010s, the methodological repertoire used for identifying repositioning candidates has expanded, especially with data science and information technology methods (bioinformatics, AI; see, e.g., [73]). With these methods, analyzing various, large, and heterogeneous datasets, as well as gaining a deeper understanding of the complex pathomechanisms of diseases and the mechanisms of action of drugs, has become possible. This development makes candidate selection much more cost-effective and faster and has higher success rates. The other factor is related to the productivity of de novo innovation, which is still fluctuating. It is only partially satisfying medical needs in several areas, requiring huge expenditures, which is, among other things, a consequence of the high failure rate of de novo innovation. Thus, somewhat paradoxically, de novo innovation also motivates the use of the repositioning strategy. Nevertheless, drug repositioning has only partially exploited these opportunities; surprisingly few repositioning drugs have been approved for human use and even fewer for veterinary use. It is also interesting that the repositioning strategy has been used much less in the veterinary area.

What are the most important tasks? A key aspect is increasing the productivity of repositioning. It does not seem sufficient to focus on a single component; rather, a “multi-targeting” approach is needed. In short, the implementation of the latest technologies, with professional and motivated actors and an efficient structure with a solid conceptual basis, is required. The participation of the for-profit and non-profit sectors is important in pharmaceutical repositioning projects, as other studies also emphasize (see, e.g., [236]). For the profit-oriented sector, the guarantees of market exclusivity do not seem strong enough, and therefore, the realization of the expected business results may also seem uncertain (see, e.g., [31]). At the same time, well-documented examples support that strong patent protection is possible for the second medical use, especially with generics and new formulations [44]. Incorrect off-label use, which can reduce the business potential of repositioning, can be prevented or at least suppressed with patent protection and regulatory interventions.

We also believe, in line with other opinions [44,236], that the wider involvement of the non-profit sector, of which universities are prominent representatives, can also support practical drug repositioning. However, difficulties may arise, above all in terms of academic innovation, especially limited knowledge and skills in intellectual property protection and in drug development. Fortunately, the academic scenario is changing. The recently established or reorganized innovation units integrated into universities, the renewed university training programs that also cover various aspects of innovation, and the involvement of the national and regional health and innovation policy authorities [237,238] have had a positive effect. As a result, many universities, including veterinary universities, have recently become more active in drug innovation, especially in the application of the repositioning strategy.

We argue that university participation is justified for several reasons. The innovation-relevant university competencies, together with their traditional research-relevant professional skills, can improve the translation of university research to innovation. Adequate preclinical tools and methods are in part also available at universities, as pharmaceutical repositioning projects do not necessarily require very expensive specialized infrastructure (such as that which companies routinely require for de novo innovation). Moreover, there can be efficient funding systems for the motivation of the involvement of universities [237]. For example, recently, a subcommittee of the EU launched a program to support the drug repositioning activities of not-for-profit organizations [238]. Finally, we also refer to the generally more flexible, creative, and holistic approach of academic scientists, which can be particularly effective for drug repositioning projects. All this does not mean, and it would be a mistake, encouraging universities to “industrialize”, nor should they abandon their complementary skills. Instead, universities should work alongside pharmaceutical companies to innovate and reposition drugs effectively. Such an approach would lead to much closer innovative, goal-oriented cooperation between the non-profit and profit-oriented sectors, optimally, in drug repositioning.

Furthermore, expanding the traditional two-part human medicine industry structure into three parts with veterinary medicine would be another significant step towards increasing repositioning productivity in both human and veterinary medicine. The professional tools and expertise of human preclinical and clinical drug development can also be utilized in veterinary drug repositioning, as confirmed by other research studies, especially in the oncology disease area. From the veterinary side, the results of veterinary drug development studies and real-world observations and data analysis could be used for the development of reliable and highly predictive preclinical in vivo models for human repositioning, instead of poorly performing series of models of de novo innovation. The synergistic structure we propose makes it possible for the two hitherto independent, often rocky, paths of human and veterinary repositioning to converge into one that may end fruitfully for both areas.

Our previous thoughts led us to the One Health approach to drug repositioning, which ultimately provides the conceptual basis of the One Health-based structure of a new platform outlined in the sixth section. We name this new platform the One Health Drug Repositioning Platform. Our scope obviously goes far beyond a simple coordinating role in a single discipline or a single disease area of human and/or veterinary drug research. However, there is no doubt that a limited scope approach is interesting and important in itself; it is noteworthy that such a strategy is under research [239]. In our opinion, the One Health approach can also be extended to de novo drug innovation. We will work on this extension in a later phase.

We believe that the positive examples of drug repositioning, presented in critical articles and reviews, as well as in this review, have potential. The proposals formulated in the publications we refer to, together with our own proposals for significantly improving performance, promise a new perspective for drug repositioning as a productive, cost-effective innovation path in both human and veterinary medicine, providing benefits for both human and animal patients and healthcare. It is our hope that this review will encourage the mutual use of achievements in human and veterinary drug innovation and stimulate interest in further exploiting the innovative potential of drug repositioning.

## Data Availability

The data presented in this study are available on request from the corresponding author.
